# Toward Value-Based Reimbursement for Hemophilia A in the UAE: A Multi-Stakeholder Perspective

**DOI:** 10.3390/jmahp14040058

**Published:** 2026-09-29

**Authors:** Sara Al Dallal, Hatem Abuieda, Nasser AlZain, Qassim Farouq, Shandhosh Raman, Sherif Mahmoud, Waiel Al Naeem, Ahmed Osman

**Affiliations:** 1 Emirates Health Economics Society, Dubai P.O. Box 6600, United Arab Emirates; 2 International Society for Pharmacoeconomics and Outcomes Research, UAE Chapter, Abu Dhabi P.O. Box 44171, United Arab Emirates; abueida2000@yahoo.de; 3 Mediclinic, Dubai P.O. Box 505004, United Arab Emirates; naseralzein@yahoo.com; 4 MetLife, Dubai P.O. Box 371916, United Arab Emirates; drqasimfarooq77@gmail.com; 5 Medical Standards and Patient Care, Department of Health, Abu Dhabi P.O. Box 5674, United Arab Emirates; sraman@doh.gov.ae; 6 Syrenia Solutions, Dubai P.O. Box 123016, United Arab Emirates; sherif.mahmoud@syreniasolutions.com; 7 Pharmacy Department, Sheikh Khalifa Medical City, Abu Dhabi P.O. Box 51900, United Arab Emirates; walnaeem@seha.ae; 8 Payers Division, Department of Health, Abu Dhabi P.O. Box 5674, United Arab Emirates; aosman@doh.gov.ae

**Keywords:** hemophilia A, emicizumab, health technology assessment, value-based healthcare, real-world evidence, reimbursement, health policy, patient-centered care, United Arab Emirates

## Abstract

Hemophilia A management in the United Arab Emirates (UAE) is still largely driven by treatment acquisition costs. This may limit access to innovative therapies such as emicizumab despite their broader clinical value. This paper aimed to identify barriers to value-based reimbursement and develop policy recommendations to strengthen health technology assessment (HTA) for hemophilia A in the UAE. An evidence-informed multi-stakeholder roundtable was held during the International Society for Pharmacoeconomics and Outcomes Research (ISPOR) UAE Chapter 2026 Conference. The discussion was informed by published evidence, regional clinical guidance, and expert perspectives. The roundtable discussion was transcribed and summarized using descriptive qualitative analysis, and the manuscript synthesizes the main policy themes identified by the participants. Four priority areas emerged: adopting value-based assessment beyond acquisition cost, strengthening the use of evidence in reimbursement decisions, establishing national hemophilia registries with standardized data collection, and improving timely access to innovative therapies for high-risk patients. Overall, participants agreed that stronger HTA processes and better evidence generation are needed to support reimbursement decisions. Implementing these recommendations could improve access to innovative therapies and support more consistent, patient-centered, and sustainable hemophilia care in the UAE.

## 1. Introduction

Hemophilia A is a rare X-linked bleeding disorder caused by mutations in the gene encoding coagulation factor VIII (FVIII) [1]. Patients often experience severe spontaneous complications, such as bleeding in the joints, muscles, and organs. Globally, hemophilia occurs in approximately 1 in 5000 males [1]. In the Middle East and North Africa, the prevalence rate is 4–8.1 cases per 100,000 population [2]. The frequency is thought to be higher in the Gulf region because of the high rates of consanguineous marriage [3]. Saudi Arabia registered 1134 cases, whereas Kuwait documented approximately 100 cases. The United Arab Emirates )UAE( lacks hemophilia A counts due to fragmented records across all emirates [3].

Traditional management involves intravenous factor VIII prophylaxis two to three times a week. This approach significantly reduces bleeding episodes compared to on-demand treatment. Despite these benefits, there are important challenges. Repeated intravenous injections may lead to venous damage over time. Furthermore, factor VIII inhibitors develop in approximately 20–30% of patients. These challenges contribute to a higher treatment burden and affect patient compliance [4].

Emicizumab represents a significant therapeutic advancement in the management of hemophilia A. It is a subcutaneously administered recombinant humanized bispecific monoclonal antibody that functionally mimics factor VIII by bridging activated factor IX and factor X, thereby promoting thrombin generation [5]. The HAVEN 1 trial demonstrated an 87% reduction in treated bleeding events compared with no prophylaxis in patients with factor VIII inhibitors [6]. Similarly, HAVEN 3 showed a 68% reduction in bleeding rates compared with factor VIII prophylaxis in patients without factor VIII inhibitors. Furthermore, HAVEN 2 and HAVEN 4 confirmed its efficacy in the pediatric population and supported its long-term effectiveness [7,8].

Real-world data from clinicians in the UAE and other countries indicate outcomes that are consistent with clinical trial observations [3]. The reported annual bleeding rate (ABR) approaches zero in many patients. Bleeding in the target joints was reduced or resolved. In addition, emergency healthcare utilization decreased by approximately 50–75%. These clinical improvements have been associated with a better quality of life, including improved school attendance and physical activity [3,9].

The adoption of innovative therapies is constrained by several health system and reimbursement challenges. Patient registries remain fragmented across entities such as the Department of Health (DoH)—Abu Dhabi, Dubai Health Authority (DHA), SEHA, and private providers. In addition, reimbursement bodies often focus primarily on acquisition costs rather than on system-wide savings. Despite the indirect costs, such as the loss of caregiver productivity, which account for 20–40% of the total disease burden, these parameters are not routinely measured. Additionally, existing health technology assessment (HTA) frameworks may not fully incorporate the broader clinical, societal, and healthcare system benefits of rare disease interventions [2,3,10]. Figure 1 presents the key structural gaps in governance, data infrastructure, clinical pathways, and reimbursement identified from published Gulf hemophilia A consensus statements and relevant policy literature. These evidence-informed findings served as the foundation for the roundtable discussion, providing a structured framework for the multi-stakeholder dialogue and subsequent policy recommendations [3,11].

This paper synthesizes the available evidence to inform practical policy recommendations to strengthen hemophilia care and improve access in the UAE. On 30 January 2026, the International Society for Pharmacoeconomics and Outcomes Research (ISPOR) UAE convened a multi-stakeholder roundtable to discuss evidence-based solutions. The discussion highlighted key gaps in data systems, clinical guidance, and value assessment that affect reimbursement decisions and access to innovative therapies. It also supports the UAE’s transition toward more evidence-based reimbursement for rare diseases.

Hemophilia A was selected as a model for policy development because of its well-defined patient population and measurable clinical outcomes. Gulf consensus statements have established clinical management pathways, and national guidance has outlined general HTA processes. However, these frameworks do not provide disease-specific recommendations for value-based reimbursement in hemophilia A. They also give limited attention to local real-world evidence (RWE), short-term budget impact, and multi-stakeholder input. This paper addresses these gaps by combining published evidence, local real-world data, and expert perspectives to propose practical recommendations for value-based reimbursement and evidence generation in the UAE.

## 2. Materials and Methods

### 2.1. Design and Settings

This paper was developed using an evidence-informed approach through a moderated roundtable at the ISPOR UAE Chapter 2026 Conference on 30 January 2026. The discussion addressed the question: how can innovative hemophilia A therapies be optimally implemented in UAE healthcare systems to support value-based management? The approach followed four sequential phases: (1) a targeted scoping review of published literature, regional policy considerations, and relevant UAE policy documents; (2) identification of key evidence and policy gaps; (3) expert validation and contextualization of these gaps through the moderated roundtable; and (4) mapping of the validated gaps and recommendations into strategic policy domains.

### 2.2. Expert Engagement

The 90-min roundtable included 8 participants in total, including a chairperson, moderator, two speakers, and 4 additional experts. Participants represented hematology, health economics, insurance, reimbursement bodies, regulatory authorities, national health authorities, and healthcare organizations.

### 2.3. Data Collection

Two technical presentations were structured for discussion. First, clinical outcomes of emicizumab in severe hemophilia A: RWE from Tawam Hospital and Sheikh Khalifa Medical City (SKMC) in the UAE. Secondly, the economic outcomes of emicizumab for the management of children and adults with hemophilia A in the UAE.

### 2.4. Discussion Structure

The discussion drew on local RWE, Gulf consensus guidelines, and expert experience. It was guided by four open-ended questions that focused on three strategic priorities: gaps in RWE infrastructure, fragmented governance that slows national coordination, and unit-cost reimbursement practices that are misaligned with the total system value (Table 1).

### 2.5. Analysis

The session was recorded, transcribed, and analyzed by two independent researchers to reduce bias. A descriptive analysis framework was used to summarize expert perspectives, areas of agreement and divergence, policy gaps, and proposed actions across the predefined strategic domains.

The summarized themes and key findings were subsequently circulated to the roundtable participants for review and confirmation prior to finalization. A structured gap assessment then compared current practices with the Gulf consensus guidelines, DoH HTA requirements, and RWE benchmarks.

### 2.6. Integration with Value-Based Framework

The analysis synthesized published evidence and expert perspectives to identify policy priorities and inform reimbursement-related recommendations within the UAE healthcare context.

## 3. Results and Discussion

### 3.1. Shifting Hemophilia A Reimbursement from Factor VIII Cost Focus to Total Value Assessment

Despite the clinical superiority of emicizumab, the discussion indicated that acquisition cost remains the primary driver of reimbursement decisions. Current approval processes still overly rely on traditional factor VIII pricing rather than a broader system-level value.

The discussion highlighted that patients do not require an inhibitor status to be eligible for emicizumab. Patients who are stable on factor VIII prophylaxis qualify if they have a high risk of bleeding or joint damage. This aligns with the Gulf region consensus guidelines recommending non-factor therapies, such as emicizumab, as standard care for moderate-to-severe hemophilia A, regardless of inhibitor development [3].

Regionally, Saudi Arabia and Kuwait recommend emicizumab prophylaxis to prevent arthropathy and hospitalization [3]. From an insurance perspective, reimbursement should follow the clinical authority guidance from the DoH hematology protocols. This approach ensures appropriate access while supporting long-term cost control. Early emicizumab use reduces the need for emergency visits, joint replacements, and productivity losses. This translates into lower overall healthcare costs while improving long-term patient outcomes [12].

Overall, the total value assessment focuses on bleeding prevention, joint health, and quality of life. This approach helps make sustainable reimbursement decisions for the treatment of rare diseases.

#### Key Strategic Recommendations

- Shift eligibility criteria from factor VIII cost comparison to clinical outcomes, including bleeding rates and joint health.
- Update reimbursement policies in line with Gulf consensus guidelines and DoH hematology protocols as the main clinical references.
- Establish total value HTA frameworks that prioritize bleeding prevention, protection of joint health, and quality of life improvement, not just drug acquisition costs.
- Introduce risk-based approval pathways to allow early access to emicizumab for patients with moderate to severe hemophilia A.

### 3.2. Clinical and Economic Evidence-Based Value Communication

From a policy perspective, an early transition to emicizumab for patients with severe hemophilia A is recommended to prevent lifelong complications. The main value of this treatment is the reduction in annual bleeding rates and preservation of joint function. Health economists have emphasized that evidence should be presented through budget impact models that capture system-wide savings beyond pharmacy budgets.

RWE from Tawam and SKMC Hospitals showed that emicizumab was associated with substantial reductions in bleeding outcomes among patients with severe hemophilia A. In the retrospective two-center cohort of 30 patients, the ABR decreased from 2.9 to 0, joint bleeds declined from 2.3 to 0, and emergency visits fell from 3.3 to 0.1 per year. Health-related quality of life (HRQoL) also improved after treatment initiation, with lower scores indicating better health status on the Haemo-QoL questionnaire for children and Haem-A-QoL for adults, as reflected by the decrease from 78.0 to 30.5 [13].

Table 2 presents an exploratory 12-month budget-impact analysis of 30 patients treated at Tawam Hospital and SKMC. Although emicizumab prophylaxis had higher acquisition costs by 3%, these were offset by lower bleeding-related and joint-bleed costs, and the model estimated a 15% reduction in total direct healthcare costs. These findings are based on a small retrospective cohort from two tertiary centers and should be considered preliminary and hypothesis-generating rather than definitive evidence of long-term economic benefit. Larger real-world studies are needed to confirm the long-term economic value of emicizumab.

Published studies have shown that emicizumab reduces annualized bleeding rates by 68–87 percent compared with factor VIII prophylaxis [6,14]. By preventing bleeding and joint complications, it is associated with substantial reductions in costs associated with surgeries, joint replacements, and emergencies. Subcutaneous administration also lowers indirect costs, such as parental absenteeism and caregiver expenses.

Overall, the findings support the clinical value of emicizumab and reinforce its role within a value-based approach to the management of hemophilia A.

#### Key Strategic Recommendations

- Move from price-only reviews to total value assessment.
- Establish DoH/DHA/hematology society coordination to ensure guideline-aligned nationwide reimbursement.
- Value-based and long-term reimbursement models should be used. Link payment to outcomes, such as fewer bleeds and fewer emergency visits.
- Adopt a risk-based reimbursement approach that enables timely access for high-risk patients, reducing joint damage, disability, and future healthcare resource utilization.
- Expanding patient support programs and shared funding for rare diseases is essential. Structured training should be provided for providers, and pharmacist education should be reimbursed to improve adherence.

### 3.3. National Registries and RWE

Experts have identified the absence of national registries as a major barrier, although UAE health-information exchange platforms, including Malaffi in Abu Dhabi and NABIDAH in Dubai, are available. Rare disease registries should be prioritized based on their cost impact. They should collect data before and after therapy to support evidence-based decision-making.

Although operational data are available, they lack clinical details, patient-reported outcomes, and HRQoL metrics essential for value assessment. Federal coordination through ministry oversight can enable data sharing across all Emirates. Integrating RWE into HTA allows evaluations to focus on the total system value rather than individual departmental budgets.

Beyond registry infrastructure, participants emphasized integrated care models that may reduce treatment burden and support patient and caregiver quality of life. Although hemophilia care is typically initiated and overseen by specialist teams; but certified general practitioners can contribute to ongoing care within defined shared-care pathways. Reimbursement bodies could support pharmacist-led education and patient-support services to strengthen long-term treatment adherence.

#### Key Strategic Recommendations

- Prioritize establishing a national hemophilia registry should support standardized longitudinal collection of clinical outcomes, healthcare utilization, and patient-reported outcomes to strengthen RWE generation and inform lifecycle HTA.
- Establish federal Ministry oversight to allow data sharing across the Emirates and address confidentiality barriers.

## 4. Limitations

The findings should be interpreted in light of the following limitations. First, the roundtable included only ten experts from healthcare organizations in Abu Dhabi, Dubai, and national institutions. Consequently, the findings may not fully reflect the perspectives of all stakeholders involved in hemophilia care across the UAE.

Second, the discussion was qualitative, which may have introduced bias and subjective views that are common in expert meetings. Third, the estimated savings and clinical outcomes were based on small two-center datasets and a short-term model. Therefore, the findings should be considered preliminary and hypothesis-generating in nature.

Fourth, the economic model focused on the budget impact for 12 months and did not include a full sensitivity analysis or long-term projections. Finally, the recommendations still need validation through federal stakeholder consultation and pilot testing to confirm that they are practical across the UAE healthcare systems.

## 5. Conclusions

In a small two-centre UAE cohort, emicizumab prophylaxis was associated with reductions in annual bleeding, joint bleeding, and emergency visits. It also results in significant cost savings of 15% over one year, based on local RWE. Despite this, UAE reimbursement still focuses on factor VIII costs rather than the overall value of the therapy. At the same time, regional consensus supports the use of non-factor prophylaxis for moderate-to-severe hemophilia A. This applies to all patients, regardless of their inhibitor status. These insights provide a strong basis for repositioning emicizumab reimbursement to align with evidence-based frameworks and local consensus.

The roundtable resulted in strong recommendations to establish national hemophilia registries capturing standardized annual bleeding rates and quality-of-life (QoL) measures before and after therapy. Participants also recommended that the Ministry of Health (MoH) serve as the federal custodian for RWE generation. Further recommendations included aligning clinical guidelines with DoH requirements to define risk-based eligibility and treatment sequencing. Multidisciplinary training programs were also recommended to strengthen the competence and confidence of hematologists, general practitioners, and pharmacists in guideline-based care.

These measures are essential to address data fragmentation and governance gaps. They can help reduce the lifelong clinical and economic burden of hemophilia in the UAE. Proper implementation will sustain access to high-value therapies, prevent joint damage, and position the UAE as a regional example of value-based care for hemophilia A and potentially other rare diseases.

## Figures and Tables

**Figure 1:**
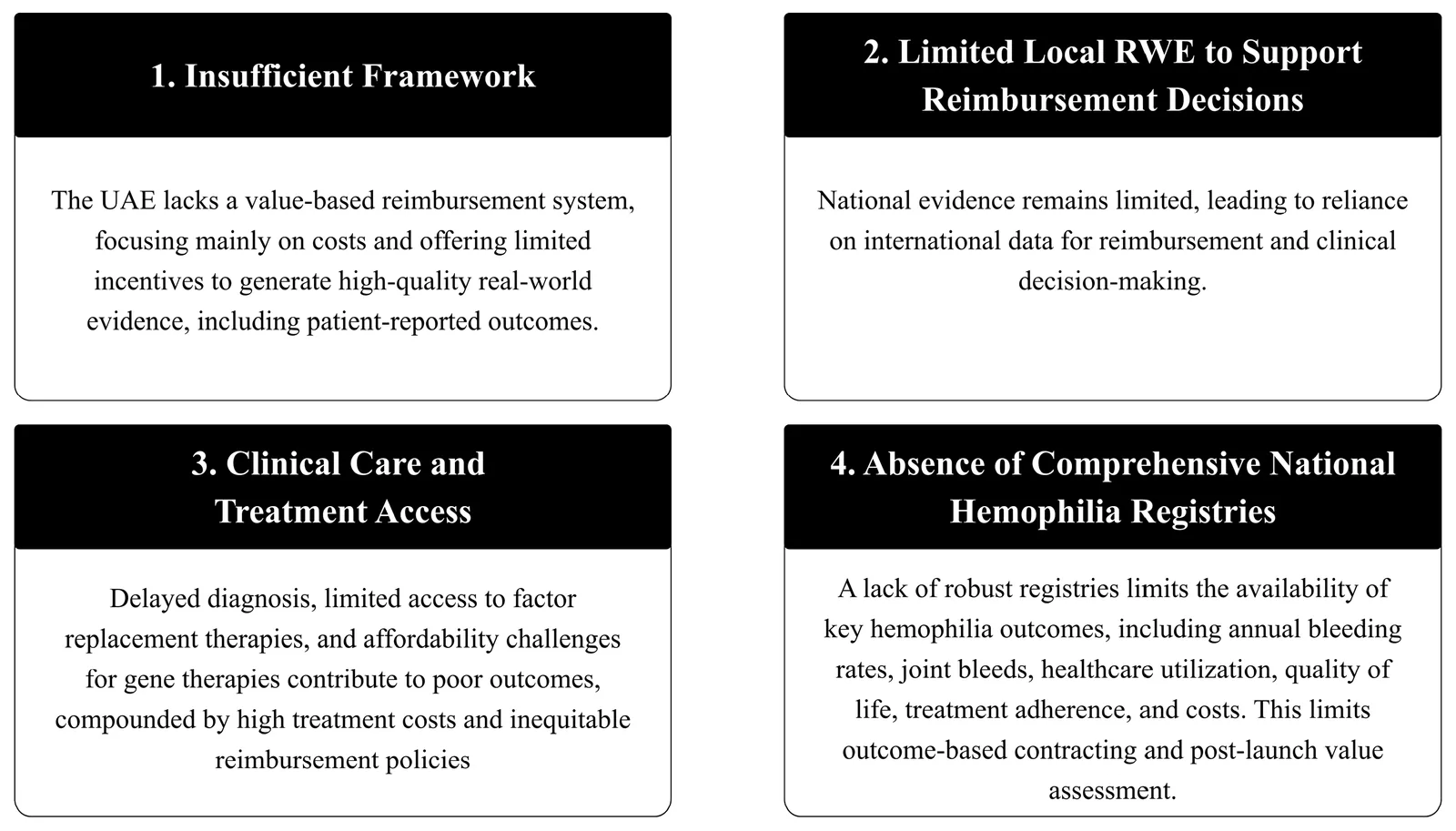
Structural Barriers to Value-Based Hemophilia A Care in the UAE. Abbreviations: RWE, Real-World Evidence; UAE, United Arab Emirates.

**Table 1:** Strategic domains driving the UAE hemophilia A policy priorities.

Domain	Areas of Interest	Policy Implications
National RWE Infrastructure	Registry gaps, data standardization, federal oversight	Enables lifecycle HTA, performance-based agreements
Clinical Guidelines & Pathways	Absent algorithms, eligibility criteria, sequencing	Aligns practice with Gulf consensus, prevents over-expansion
Total-Value Reimbursement	Unit-cost vs. system savings, direct/indirect costs	Cost savings realization, sustainable rare disease access

**Table 2:** Budget impact analysis of emicizumab prophylaxis vs. factor VIII.

Cost Category	Pre-Emicizumab (AED, Cohort/Year) *	Post-Emicizumab (AED, Cohort/Year)	Savings
Prophylaxis	45.4M *	46.9M	3% (increase)
Bleeding Events	0.85M	0.01M	99%
Joint Bleeds	8.8M	0	≈100%
Total Direct Cost	55.4M	47.2M	15%

* AED: United Arab Emirates dirham, M: Millions.

## Data Availability

No new data were created or analyzed in this study. Data sharing is not applicable to this article.

## Abbreviations

The following abbreviations are used in this manuscript:

ABR	Annual bleeding rate
AED	United Arab Emirates dirham
DHA	Dubai Health Authority
DoH	Department of Health, Abu Dhabi
HRQoL	Health-related quality of life
HTA	Health Technology Assessment
ISPOR	International Society for Pharmacoeconomics and Outcomes Research
MoH	Ministry of Health
QoL	Quality of life
RWE	Real-world evidence
SEHA	Abu Dhabi Health Services Company
SKMC	Sheikh Khalifa Medical City
UAE	United Arab Emirates
